# Editorial: Molecular mechanisms of resistance to “last resort” antimicrobials in Enterobacterales

**DOI:** 10.3389/fcimb.2024.1429200

**Published:** 2024-05-30

**Authors:** John Osei Sekyere, Thamarai Schneiders, Piotr Majewski

**Affiliations:** ^1^ Medical Diagnostic Laboratories, Institute of Biomarker Research, Department of Clinical Development, Genesis Biotechnology Group, Hamilton Township, NJ, United States; ^2^ Department of Dermatology, School of Medicine, Faculty of Health Sciences, University of Pretoria, Pretoria, South Africa; ^3^ Center for Inflammation Research, Institute of Regeneration and Repair, University of Edinburgh, Edinburgh, United Kingdom; ^4^ Department of Microbiological Diagnostics and Infectious Immunology, Medical University of Białystok, Białystok, Poland

**Keywords:** colistin, tigecycline, carbapenem, reserved antibiotics, last-resort antibiotics, Enterobacterales

The use and misuse of antibiotics in veterinary and human medicine, agriculture, and aquaculture are selecting resistance genes in different bacterial species (Osei Sekyere, 2016; Tang et al., 2017) Owing to this selection pressure, bacterial strains with resistance to several, if not all antibiotics in current use (also known as pandrug resistance), are emerging (Manageiro et al., 2012; Guducuoglu et al., 2018; Kopotsa et al., 2020; Osei Sekyere and Reta, 2020). Further, more and more of these resistance genes are escaping the chromosome unto mobile genetic elements (MGEs) i.e., plasmids, transposons, integrons, and integrative conjugative elements, under antibiotic pressure, enabling erstwhile susceptible strains to horizontally obtain these mobile resistance genes (Pedersen et al., 2018; Kopotsa et al., 2019, 2020). This worrying trend presents a great challenge for human medicine as recommended antibiotics become ineffective against common bacterial pathogens (Manageiro et al., 2012; Guducuoglu et al., 2018). The emergence of carbapenem, colistin, and tigecycline resistance is a perfect example of this phenomenon of antibiotic resistance evolution (Osei Sekyere et al., 2016).

Specifically, the use of extended-spectrum penicillins and cephalosporins to treat penicillin-resistant infections, mediated by AmpCs, bla_OXA_, *bla*_SHV_ and *bla*_TEM_, led to the selection and evolution of *bla*_CTX-M_ and plasmid-mediated AmpCs, *bla*_SHV_ and *bla*_TEM_ (Bush, 2018). With the proliferation of the penicillinases and cephalosporinases, particularly through IncF plasmids and other MGEs, carbapenems were introduced to treat infections that were insensitive to the penicillins and cephalosporins, leading to the evolution/emergence and rapid dissemination of the carbapenemases: *bla*_KPC_, *bla*_GES_, *bla*_SME_, *bla*_IMP_, *bla*_OXA-48-like_ *bla*_VIM_, *bla*_SPM_, and *bla*_NDM_ (Osei Sekyere et al., 2015; Bush, 2018; Ragheb et al., 2022). Expectedly, the carbapenemases made carbapenems, which used to be the reserved or last-resort antibiotic, ineffective against infections caused by carbapenemase-producing pathogens. Clinicians therefore resorted to colistin and tigecycline as additional reserved/last-resort antibiotics to replace or supplement carbapenem therapy in multidrug-resistant infections (Osei Sekyere et al., 2016).

In Tembisa, South Africa, an outbreak of NDM and OXA-48-like carbapenemase-producing Enterobacterales (*Klebsiella pneumoniae, Enterobacter cloacae*, and *Escherichia coli*) claimed 10 infant lives between December 2019 and March 2020 (Osei Sekyere et al.). These pathogens harbored *bla*_OXA-48-like_ *and bla*_NDM_ on plasmids and composite transposons, facilitating the spread of the carbapenemases across different species. Similarly in Bahrain (Shahid et al), the same carbapenemases were identified in 24 pandrug-resistant *K. pneumoniae*, with *bla*_NDM_ being found on IS*Aba125*. As observed in the South African strains (Osei Sekyere et al.), these 24 strains were resistant to most antibiotics including colistin (75%), ceftolozone-tazobactam (100%), piperacillin-tazobactam (96%), meropenem (92%) and intermediate resistant to tigecycline (44%).

Ceftazidime-avibactam resistance, a last-resort antibiotic reserved for multidrug-resistant *Pseudomonas aeruginosa*, was identified by Flury et al. in Switzerland in imipenem-resistant *P. aeruginosa*. These strains had truncated or absent *OprD* genes, derepressed AmpCs, and overexpressed *mexAB* and *bla*_PER-1_. As well, Li et al., isolated *Pseudomonas asiatica* strains with resistance to tigecycline and carbapenems in a hospital’s sewage in China, suggesting the presence of these strains in the hospital environment. Notably, the *P. asiatica* strains had multiple resistance genes on novel Tn*7389* and Tn*7493* transposons on large plasmids (~199, 972 bp) that were transferable to *P. aeruginosa* (but not to *E. coli*), albeit with a fitness cost to pathogenicity when tested in a *Galleria mellonella* infection model.

The clinical effect of these carbapenemases is described by Larcher et al. They assessed the effect of last-resort antibiotics such as ceftazidime-avibactam plus aztreonam, imipenem-cilastatin-relebactam, and cefiderocol (a 5th or 6th-generation cephalosporin approved in 2019) on *bla*_NDM_ -positive infections in patients hospitalized at a tertiary hospital in Nimes, France. Between 2020–2022, the infection survival rate was 45.4%, clinical failure rate was 30%, microbiological failure rate was 33%, and mortality rate was 23%. These statistics show the clinical difficulty associated with managing carbapenem-resistant infections.

Although colistin resistance is relatively recent, Mmatli et al. showed that it has spread globally, with the use of colistin-growth promoters facilitating its spread among food-producing animals, farm environments, wastewater, and ultimately, in humans. The *mcr* genes were mainly located on IncH, IncC, IncI, IncX, and IncP plasmids as well as on IS*1595*. Furthermore, Sato et al. isolated Enterobacterales from 258 companion animals in Japan and found 12 and one colistin-resistant *Enterobacter cloacae* and *K. pneumoniae* isolates, respectively. The 12 *E. cloacae* strains belonged to the same lineage as that of human-associated lineages, suggesting a potential animal-human transmission of these strains. The evolution, genetic environment, diversity and proliferation of *mcr* and *mcr* variants were further corroborated by Gaballa et al. who identified 125 putative new *mcr-like* genes from 69,814 MCR-like proteins present in 256 bacterial genera.

Tigecycline resistance, which is still limited in geographic and species spread, were reviewed by Korczak et al., who found efflux pumps, porins (in the outer membrane), regulators of efflux and porins, and *tet* genes as major tigecycline resistance mechanisms. Some of these genes were found on plasmids, which can facilitate their quick dissemination across species and wider geographic regions if tigecycline use is not well regulated.

Hence, it is evident from this Research Topic that bacterial strains that are resistant to last-resort antibiotics are being selected and spread globally, resulting in outbreaks and fatal infections. The need for continued genomic surveillance and strict antibiotic stewardship to protect these reserved antibiotics cannot be overemphasized.

## Author contributions

JO: Conceptualization, Data curation, Formal analysis, Investigation, Methodology, Project administration, Resources, Software, Supervision, Validation, Visualization, Writing – original draft, Writing – review & editing. TS: Conceptualization, Data curation, Formal analysis, Investigation, Methodology, Project administration, Resources, Software, Supervision, Validation, Visualization, Writing – review & editing. PM: Conceptualization, Data curation, Formal analysis, Investigation, Methodology, Project administration, Resources, Software, Supervision, Validation, Visualization, Writing – review & editing.
